# Swiss Odontological Society

**Published:** 1891-02

**Authors:** Theo. Frick

**Affiliations:** Zurich, Switzerland


### SWISS ODONTOLOGICAL SOCIETY.

The Fifth Annual Meeting of the Swiss Odontological Society was held at Geneva, October 4, 5, and 6, 1890.

The meeting taking place unusually late in the season, not as many dentists were present as might have been; yet there were over fifty. The president of the Society, Professor Dr. Redard (Geneva), delivered a short address, after which Dr. Magitot, the well-known French writer, read a paper on "Mutilations Ethniques," in which he spoke of the peculiar manner in which some of the non-civilized peoples mutilate their teeth, by cutting them into certain very strange shapes, giving, for instance, to all incisors the form of cuspids. The paper was listened to with great interest, yet no discussion followed, as it was of little scientific or practical value.

The second paper was read by Dr. Oltramore, of Buenos Ayres. He described his new method of making fillings of cement and gold. He prepares beforehand a piece of gold of the size and approximate shape of the opening of the cavity, by condensing a few cohesive cylinders on a serrated steel plate. He then fills two-thirds of the cavity with an oxyphosphate cement, places his prepared piece of gold on the cement while it is yet soft, and finishes the filling by putting on more cohesive gold, until the desired fulness is arrived at. Dr. Oltramore claims to have good results with these fillings and to save a great deal of labor. Time will test this method.

The third paper was read by Dr. Theo. Frick, of Zurich, "On the Treatment of Approximal Cavities in Molars and Bicuspids." The essayist had examined mouths of patients of over forty different dentists, and finds that thousands of "flat fillings" are still being made, which fail entirely to save teeth for any considerable length of time. The principal aim of his paper is, therefore, to prove that contour fillings are the only ones that will save teeth permanently. After stating that the only direct cause of caries is decomposition of food lying between the teeth, he shows how a filling must be shaped in order to prevent the food from lodging in its surroundings, and to give at the same time an uninterrupted surface for mastication. In the second part of the paper the writer describes the different steps and manipulations in making approximal fillings, calling particular attention to the separation of teeth, not with the file, but by pressure; to get a direct channel of approach to the deeper parts of the cavity; to use a thin matrix; to finish fillings by working from the labial and lingual side (with Meriam's files and other instruments). In filling difficult cavities the essayist finds great satisfaction in using a hard, dry amalgam for the cervical portion, and finishing with cohesive gold at the same sitting. He denounces the use of cements as a would-be permanent material for approximal fillings. In concluding, he hopes that more and more members of the profession will become "contourists."

Next day, Sunday, October 5, a business meeting was held, in which Dr. T. Billeter, professor of dental pathology and therapeutics at the University of Zurich, was elected president of the Society. It was also decided that, in the near future, a quarterly dental journal should be published as the official organ of the Swiss Odontological Society. The afternoon was devoted to social gatherings.

Monday was the day for clinics.

Professor Dr. Redard (Geneva) extracted teeth, producing local anæsthesia by applying ethyl chloride ($C_2H_5Cl$) as a spray on the cheek and on the mucous membrane around the tooth to be extracted.

Dr. Witzig (Bâle) demonstrated several new inventions of Dr. Herbst, of Bremen (whom he had visited a few weeks before). He called attention to his new method of making glass fillings and his method of producing an amalgam filling of good color and edge-strength, by combining it with pure silver-foil when mixing with mercury.

Dr. Oltramore (Buenos Ayres) filled several teeth (out of the mouth) by the method described in his paper.

Dr. Reverdin (Geneva) showed several new instruments, invented by him,—a very practical mouth-opener; an instrument for drawing jaw and tongue forward during narcosis with chloroform or ether; a special needle and holder for manoplastic operations.

Dr. Emery (Geneva) exhibited an instrument for extracting lower molars, which combines the advantages of the forceps and the old-fashioned key.

Dr. Kummer (Geneva) presented several patients, on whom operations like resection of the upper maxillary bone had been performed. The question how to replace the lost part artificially was discussed.

Dr. Roussy (Geneva) showed his apparatus, after Paul Bert, to give nitrous oxide gas in combination with compressed air.

Dr. Préterre (Paris) exhibited his fine collection of abnormally-shaped teeth, supernumeraries, etc., and several beautifully-made obturators.

Several other demonstrations and short communications were made. The meeting was altogether very interesting. It was decided that the next meeting should be held at Berne in the month of May, 1891.

THEO. FRICK, D.D.S.

ZURICH, SWITZERLAND.

